# GSK-J4, a Specific Histone Lysine Demethylase 6A Inhibitor, Ameliorates Lipotoxicity to Cardiomyocytes *via* Preserving H3K27 Methylation and Reducing Ferroptosis

**DOI:** 10.3389/fcvm.2022.907747

**Published:** 2022-06-02

**Authors:** Kai Xu, Xiang Liu, Bin Wen, Yazhou Liu, Wei Zhang, Xiaolin Hu, Ling Chen, Weijian Hang, Juan Chen

**Affiliations:** ^1^Division of Cardiology, Department of Internal Medicine, Tongji Hospital, Tongji Medical College, Huazhong University of Science and Technology, Wuhan, China; ^2^Department of Biochemistry and Molecular Biology, School of Basic Medicine and the Collaborative Innovation Center for Brain Science, Tongji Medical College, Huazhong University of Science and Technology, Wuhan, China; ^3^Hubei Key Laboratory of Genetics and Molecular Mechanisms of Cardiological Disorders, Wuhan, China; ^4^Neonatal Intensive Care Unit, Department of Pediatric, Tongji Hospital, Tongji Medical College, Huazhong University of Science and Technology, Wuhan, China; ^5^Department of Laboratory Medicine, Hubei University of Chinese Medicine, Wuhan, China

**Keywords:** GSK-J4, lipotoxicity, epigenetic, diabetic cardiomyocyte injury, H3K27me3, ferroptosis

## Abstract

Changes in modern lifestyle provoke a series of metabolic stresses such as hyperlipidemia. Excessive free fatty acids induce cardiomyocyte metabolic reprogramming and rearrangement of the lipid content of cardiomyocyte and promote oxidative stress. As a newly defined lipid peroxidation-related cell death pathway, the role of ferroptosis in metabolic stress-induced cardiomyocyte injury is poorly revealed. Our work indicates that GSK-J4, a histone lysine demethylase 6A/6B dual inhibitor, can alleviate palmitic acid (PA)-induced hypersensitivity to ferroptosis by suppressing H3K27 demethylation. Mechanistically, PA stimulation reduces the H3K27me3 level and hence promotes the expression of ACSL4, a key lipid modulator of ferroptosis. GSK-J4 pretreatment significantly preserves the H3K27me3 level and reduces the ACSL4 level. GSK-J4 also reduces reactive oxygen species to alleviate oxidative stress, which further decreases lipid peroxidation. Taken together, our data suggest that cardiomyocyte undergoes epigenetic reprogramming under metabolic challenges, rearranging lipid content, and sensitizing to ferroptosis. GSK-J4 can be a potential drug for treating hyperlipidemia-induced cardiomyocyte injury by targeting epigenetic modulations.

## Graphical Abstract

KDM6A is upregulated and then demethylases H3K27me3 under PA stimulation, which promotes the transcription of ACSL4 and induces ferroptosis. GSK-J4 is a specific inhibitor of KDM6A and preserves H3K27me3 under PA stimulation to suppress ACSL4 transcription, which ultimately inhibits the development of PA-induced ferroptosis and exhibits a cardiomyocyte protective effect. 
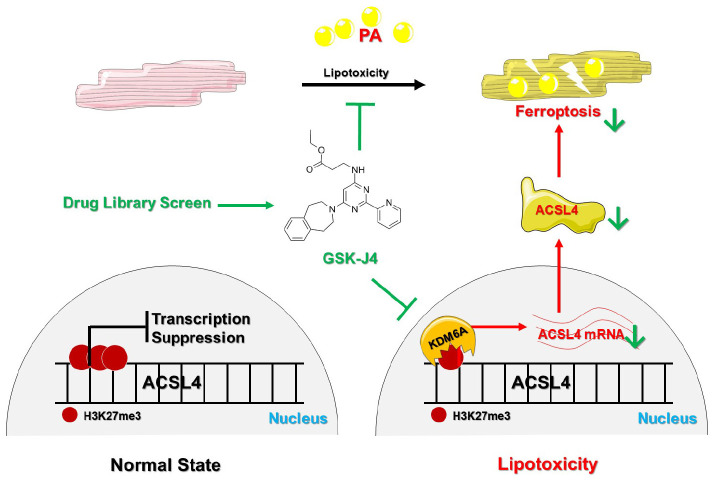

## Introduction

Cardiovascular complications account for the majority of diabetic complications and cause great burdens to the global healthcare system. Diabetes consists of two major subtypes, type 1 and type 2, where type 2 diabetes (T2D) makes up about 90% of the cases (1). The outstanding character of T2D is the systemic metabolic disturbances including elevated blood glucose and lipid content level (2, 3). Glucose toxicity (4, 5) and lipid toxicity (6, 7) are the two major metabolic insults to those energy-demanding organs such as the heart. We have previously reported that high-glucose environment could significantly induce cardiomyocyte hypertrophy by disturbing mitophagy, indicating the metabolic involvement of cardiac complications of T2D (8). However, cardiomyocyte usually takes lipid (fatty acids) as the preferential energy supplement, which suggests the contribution of lipotoxicity in the development of cardiac complications of T2D. Various reports have already shown that lipotoxicity shapes the vulnerability of cardiomyocytes to pathological stimulations both *in vitro* and *in vivo* (9, 10). These evidence lead to investigations of lipotoxicity in cardiomyocyte injuries.

One of the major problems of treating cardiac complications of T2D is that limited benefits are gained even though the metabolic disturbances are adjusted (11, 12). Evidences are also accumulating that primary cells separating from patients with diabetes show a slower proliferation rate and worse cell viability (13, 14). These clues raise the possibility that an altered metabolic environment will cause changes in epigenetic markers and further influence downstream gene expression. Epigenetic markers can alter gene expression levels without changing their sequence by recruiting different “reader” proteins to the transcription start sites or promoter region (15). By far, various kinds of epigenetic modifications are identified, such as DNA methylation, histone methylation, acetylation, phosphorylation, and so on (16). Different modifications at different sites can cause opposite downstream effects.

The effect of epigenetic modification has been widely studied in the field of cancer biology, reproductive medicine, and developmental biology. However, only a few of researches have revealed its role in the cardiovascular system. Hussain et al. demonstrated that hyperglycemia could induce myocardial dysfunction *via* epigenetic regulation of JunD, an important gatekeeper against oxidative stress (17). Olaniyi et al. showed that suppressing histone deacetylase (HDAC), an important “eraser” of histone acetylation modification, could rectify cardiac metabolic disturbance in diabetic rats (18). These results indicate that epigenetic regulation is indeed involved in the pathophysiological process of cardiac injuries caused by T2D, and suggest that targeting epigenetic modification is a promising way to treat diabetic cardiac complications.

Lipotoxicity can cause cardiomyocyte death *via* different mechanisms such as endoplasmic reticulum stress (ERS)-induced apoptosis, mitochondria dysfunction, and newly defined ferroptosis (19). Ferroptosis is characterized as lipid peroxidation which is a natural consequence of lipotoxicity (20). In doxorubicin-induced cardiomyopathy, excessive lipid peroxidation products induce mitochondrial dysfunction and subsequent ferroptosis (21). Recently, ferroptosis is found to be involved in the development of diabetic cardiomyopathy and is a promising interfering target (20, 22). Several reports have already indicated that epigenetics is involved in regulating ferroptosis (23, 24). However, whether epigenetic modifications are involved in ferroptosis in diabetic cardiomyocyte injuries is not clear.

In this research, we found that GSK-J4 showed protective effects against palmitic acid (PA)-induced cardiomyocyte injury by screening an ERS-related small molecule drug library. As a specific inhibitor of histone lysine demethylase 6A (KDM6A) inhibitor, the following studies proved that GSK-J4 exhibited a protective effect by upregulating H3K27 trimethylation (H3K27me3) and reducing the sensitivity of cardiomyocyte to ferroptosis.

## Results

### GSK-J4 Reduced Palmitic Acid-Induced Cardiomyocyte Injuries by Attenuating Endoplasmic Reticulum Stress, but Not Ameliorating Apoptosis

**FIGURE 1:**
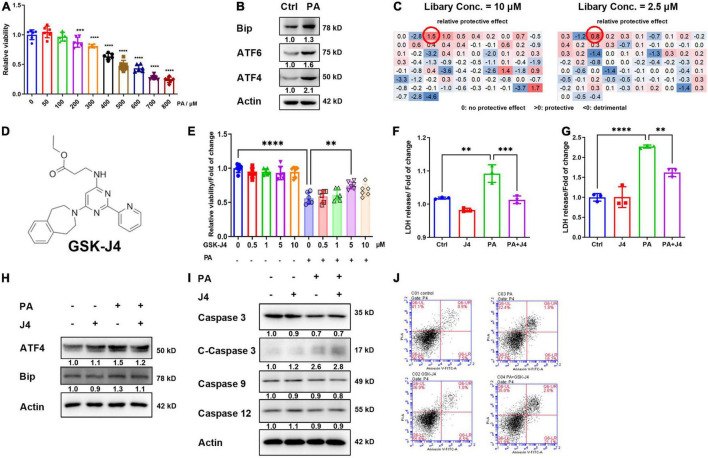
GSK-J4 ameliorates PA-induced cardiomyocyte injury independent from apoptosis. **(A)** PA dose-dependently reduces cell viability. **(B)** Significant ERS is induced by 400 μM PA stimulation for 24 h. **(C,D)** Screen potential protective drugs by cell viability with ERS-related drug screen library (MCE, New Jersey, United States, HY-L054, ERS Library, HYCPK8142) at two different concentrations **(C)** and found that GSK-J4 shows a significant protective effect at both concentrations. The molecular structure of GSK-J4 is shown in **(D). (E)** Confirm the screen result by CCK-8, and GSK-J4 indeed has a protective effect at 5 μM concentration. **(F)** PA induces LDH release from AC16 cells, while GSK-J4 reduces LDH release. **(G)** PA induces LDH release from NRCM, while GSK-J4 reduces LDH release. **(H)** GSK-J4 attenuates PA-induced ERS. **(I,J)** GSK-J4 does not ameliorate PA-induced cell apoptosis as evidenced by no altered caspases **(I)** and Annexin V/PI double staining **(J)**. ^**^P < 0.01, ^***^P < 0.001, ^****^P < 0.0001, all the data significance was analyzed by ANOVA.

We first established the PA-induced injury model by AC16 human cardiomyocyte cell lines and found that the cell viability of AC16 cells showed a dose-dependent reduction with PA concentration, and 400 μM PA caused about a 40% decrease in cell viability (Figure 1A). Western blot analysis of classical ERS marker genes such as Bip, ATF4, and ATF6 also indicated the occurrence of ERS (Figure 1B), which was also consistent with former reports.

Hence, we applied a commercial small molecule drug screening library targeting ERS, and found that GSK-J4 showed an excellent protective effect against PA-induced cell injury at low (2.5 μM) and high (10 μM) concentrations (Figures 1C,D). To confirm the result from the screen, we conducted a confirmative experiment by CCK-8 cell viability analysis and found that GKS-J4 showed a dose-dependent protective effect against PA-induced cell injury (Figure 1E). Cardiomyocytes will release lactate dehydratase (LDH) into the medium once they get damaged, making LDH an useful index for evaluating cardiomyocyte injury (25). Hence, we analyzed the LDH level in the culture medium of AC16 cell (Figure 1F) and neonatal rat cardiomyocyte (NRCM) (Figure 1G) and found that PA could stimulate LDH release, while GSK-J4 significantly reduced its releasement. These data indicated that GSK-J4 could protect cardiomyocytes from PA-induced damage. GSK-K4 could indeed attenuate PA-triggered ERS (Figure 1H). Previous reports indicated that prolonged ERS could trigger apoptosis. Hence, we exanimated the effect of GSK-J4 on apoptosis. To our surprise, although GSK-J4 showed an impressive protective effect, it seemed that it did not affect apoptosis, since no reverse of caspases (Figure 1I) and annexin V/PI double staining (Figure 1J) could be observed. These results indicated that GSK-J4 may exhibit a cardiomyocyte protective effect *via* other mechanisms.

### GSK-J4 Targeted at Histone Lysine Demethylase 6A and Preserved H3K27me3 Level Under Palmitic Acid Stimulation

**FIGURE 2:**
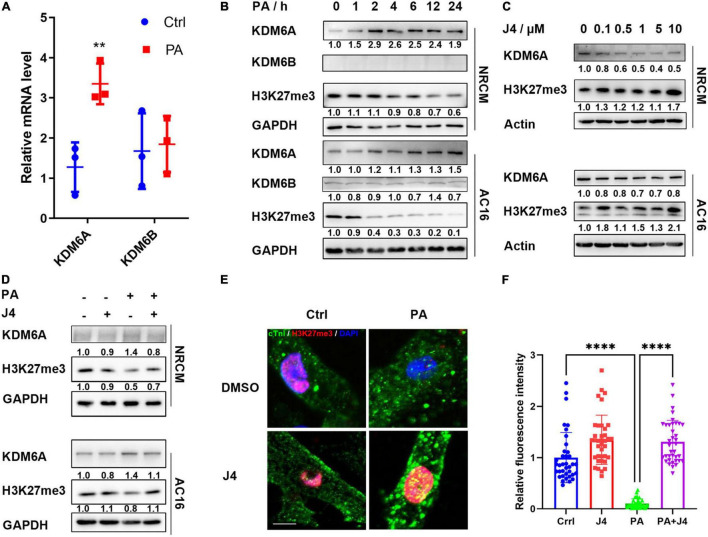
GSK-J4 inhibits KDM6A and preserves H3K27me3 level under PA stimulation. **(A)** Real-time PCR shows the mRNA level of KDM6A and KDM6B after PA stimulation. **(B)** KDM6A is gradually upregulated, while H3K27me3 is downregulated after PA stimulation both in NRCM and AC16, and KDM6B shows little difference. **(C)** GSK-J4 dose-dependently reduces KDM6A and H3K27me3 levels. **(D–F)** Pretreating NRCM or AC16 with GSK-J4 for 2 h preserves the H3K27me3 level, as evidenced by Western blot **(D)** and immunofluorescence **(E)**, the scale bar is 5 μm, and the quantification of fluorescence intensity is shown in **(F)**. ^**^P < 0.01, ^****^P < 0.0001, all the data significance was analyzed by ANOVA.

GSK-J4 is a specific dual antagonist against histone lysine demethylase 6A/6B (KDM6A/6B) and was reported to be effective in treating diabetic nephropathy (26). KDM6A/6B is correspondent H3K27me3 “eraser,” reducing its methylation level. H3K27me3 is considered a transcriptional repressive marker and could inhibit target gene expression by condensing chromatin (27). We first examined the mRNA level of KDM6A/6B and found that only KDM6A was upregulated by PA stimulation (Figure 2A). Next, we found that KDM6A showed time-dependent upregulation after PA stimulation both in NRCM and AC16 cell lines, while KDM6B did not express in NRCM and showed no time-dependent increase after PA stimulation in AC16 cell lines (Figure 2B). Taking these together, we confirmed that GSK-J4 showed a protective effect by targeting KDM6A.

Next, we found that GSK-J4 could dose-dependently upregulate the level of H3K27me3, as evidenced by Western blot in both NRCM and AC16 cell lines (Figure 2C). GSK-J4 significantly reversed PA-induced H3K27 demethylation in NRCM and AC16 cell lines (Figure 2D), and the immunofluorescence image also indicated a similar result (Figures 2E,F). These results suggested that GSK-J4 targeted at KDM6A and preserved H3K27me3 level under PA stimulation.

### GSK-J4 Attenuated Intracellular Triglyceride Accumulation

**FIGURE 3:**
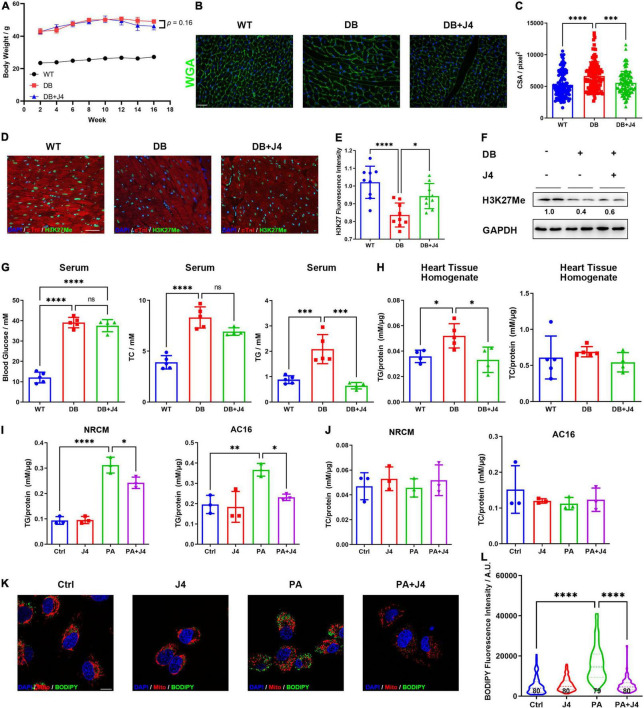
GSK-J4 reduces intracellular triglyceride accumulation and global metabolic disorder *in vivo* and *in vitro*. **(A)** Bodyweight change of DB/DB mice and WT control mice in 16 weeks. DB/DB mice were given GSK-J4 every 2 days intraperitoneally, and body weight is recorded every week. **(B)** WGA staining of mice cardiac tissue section to reflect its cross-section area (CSA), the scale bar is 20 μm. **(C)** Analysis of CSA of cardiomyocytes of mice. The CSA of DB/DB is significantly bigger than that of WT mice, and GSK-J4 treatment ameliorates the increase of CSA. **(D,E)** Typical immunofluorescence image of mice heart section staining for H3K27me3. The scale bar is 20 μm and the quantification result is shown in **(E)**. **(F)** Representative Western blot image of mice heart tissue to measure the H3K27me3 level in cardiac tissue. **(G)** Blood glucose, triglyceride, and cholesterol level in serμM. Triglyceride level is significantly downregulated, while little change is found in blood glucose and cholesterol. **(H)** Quantification of triglyceride and cholesterol levels in cardiac tissue reveals a significant reduction of tissue triglyceride levels. **(I)** Intracellular triglyceride level is upregulated by PA stimulation and GSK-J4 reduces intracellular triglyceride level. **(J)** PA stimulation shows little influence on intracellular cholesterol levels. **(K,L)** BODIPY staining shows more lipid droplets in AC16 cells and J4 reduces lipid droplet **(L)**, the scale bar is 10 μm. *P < 0.05, ^**^P < 0.01, ^***^P < 0.001, ^****^P < 0.0001, all the data significance was analyzed by ANOVA.

Next, we tested whether GSK-J4 showed a protective effect *in vivo*. As a classical animal model for T2D, DB/DB mice showed significantly higher body weight to wild-type control (WT). After administrating GSK-J4 for 16 weeks, the body weight showed a tendency to decrease (*p* = 0.16) (Figure 3A). Meanwhile, cardiomyocyte hypertrophy is often observed in various heart diseases, and we found that GSK-J4 could attenuate cardiomyocyte hypertrophy (Figures 3B,C), indicating that it showed a cardiac protective effect in DB/DB mice. Meanwhile, the H3K27me3 level was significantly downregulated in DB/DB mice, mimicking the phenomenon observed *in vitro*, and GSK-J4 could reverse H3K27me3 downregulation (Figures 3D–F). These results indicated that GSK-J4 may be effective *in vivo* as well.

We next examined the metabolic index such as blood glucose, serum triglyceride, and serum cholesterol levels (Figure 3G). It is impressive that GSK-J4 could significantly reverse the level of serum triglyceride of DB/DB mice, while it showed little effect on blood glucose or serum cholesterol level. Triglyceride was also accumulated in the heart tissue of DB/DB mice, and GSK-J4 attenuated its accumulation (Figure 3H). We also confirmed that PA stimulation could lead to triglyceride accumulation in NRCM and AC16 cell lines by triglyceride assay kit (Figure 3I), while it had little influence on intracellular cholesterol level (Figure 3J). Similar to *in vivo* results, GSK-J4 also reduced intracellular triglyceride levels *in vitro* (Figure 3I). Accumulated triglyceride will form more lipid droplets, which can be stained by BODIPY. Hence, we exanimated the number of lipid droplets and found that PA induced lipid droplet accumulation while GSK-J4 reduced them (Figures 3K,L).

Together, these data indicated that GSK-J4 could reduce intracellular triglyceride accumulation both *in vivo* and *in vitro*.

### GSK-J4 Ameliorated Oxidative Stress and Reduced Cell Sensitivity to Ferroptosis Triggered by Palmitic Acid Stimulation

**FIGURE 4:**
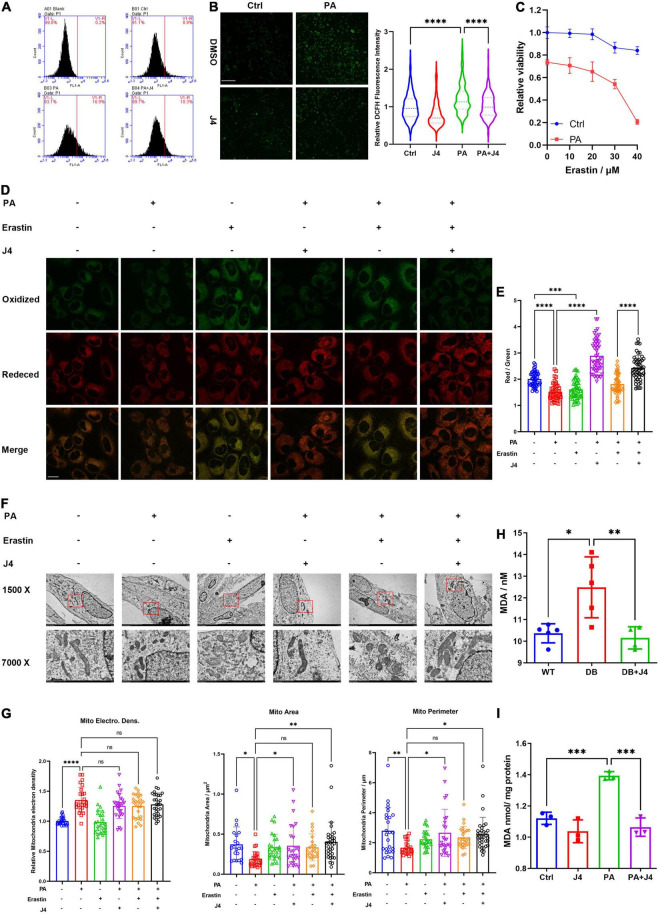
GSK-J4 inhibits PA-induced ferroptosis. **(A)** Using flow cytometric to analyze the fluorescence intensity of ROS probe DHE in the cell. PA significantly augments ROS production while GSK-J4 reduces intracellular ROS levels. **(B)** DCFH staining shows less intracellular ROS after GSK-J4 treatment, the scale bar is 50 μm. **(C)** PA amplifies the reduction of cell viability by erastin treatment. **(D)** Representative confocal image of C11-BODIPY staining of AC16 cell, where green fluorescence signal represents peroxidative lipid. **(E)** The ratio of fluorescence intensity of the red signal (reduced lipid) to green signal (peroxidative lipid). PA significantly reduced the ratio, while GSK-J4 reversed the ratio, the scale bar is 10 μm. **(F)** Representative transmission electrical microscopy image of AC16 cells with intended treatments. The mitochondrial electric density, mitochondrial area, and mitochondrial perimeter are analyzed in **(G)**. **(H)** Serum MDA level of animal models. **(I)** MDA level of cell pellet treated by PA or GSK-J4. *P < 0.05, ^**^P < 0.01, ^***^P < 0.001, ^****^P < 0.0001, all the data significance was analyzed by ANOVA.

The excessive fatty acid can provoke oxidative stress and injury cells by the production of reactive oxygen species (ROS). ROS will attack lipids on the cell membrane and cause lipid peroxidation. We found that PA could significantly induce ROS production in AC16 cell lines, while GSK-J4 could reduce ROS production by DHE flow cytometric examination (Figure 4A) and DCFH fluorescence staining (Figure 4B). These data suggested that PA could both upregulate intracellular lipid accumulation as well as ROS production, which set us thinking whether PA could provoke lipid peroxidation.

Ferroptosis is a newly defined cell death mode with the outstanding character of iron-mediated lipid peroxidation and ultimate cell death. Erastin is a classical inducer of ferroptosis (28). We found that PA could significantly provoke cell sensitivity to erastin (Figure 4C). C11-BODIPY is a sensitive lipid peroxidation probe and can detect peroxidative lipid. Once the lipid is peroxidative, the fluorescence emission light will transfer from red to green (29). Hence, the red to green fluorescence ratio can be a sensitive index to evaluate the level of ferroptosis. As shown in Figures 4D,E, PA, as well as erastin, decreased the red/green ratio, while GSK-J4 could reserve the ratio.

Another important feature of ferroptosis is the morphological damage to mitochondria. We found that PA could induce the condense of mitochondria electrical density (Figures 4F,G), but GSK-J4 did not reverse this phenomenon. However, GSK-J4 significantly reversed the over-fission of mitochondria and preserved the normal morphological features of mitochondria, for example, longer mitochondria and clear cristae. We also found that an elevated level of malondialdehyde (MDA), a byproduct of lipid peroxidation and marker of ferroptosis, was elevated both *in vivo* (Figure 4H) and *in vitro* (Figure 4I) and GSK-J4 could reverse its upregulation.

Taking together, these data indicated that GSK-J4 exhibited a cardiomyocyte protective effect partially by reducing ferroptosis triggered by PA.

### GSK-J4 Inhibited Histone Lysine Demethylase 6A and ACSL4 Expressions to Reduce Palmitic Acid-Triggered Cardiomyocyte Injury

**FIGURE 5:**
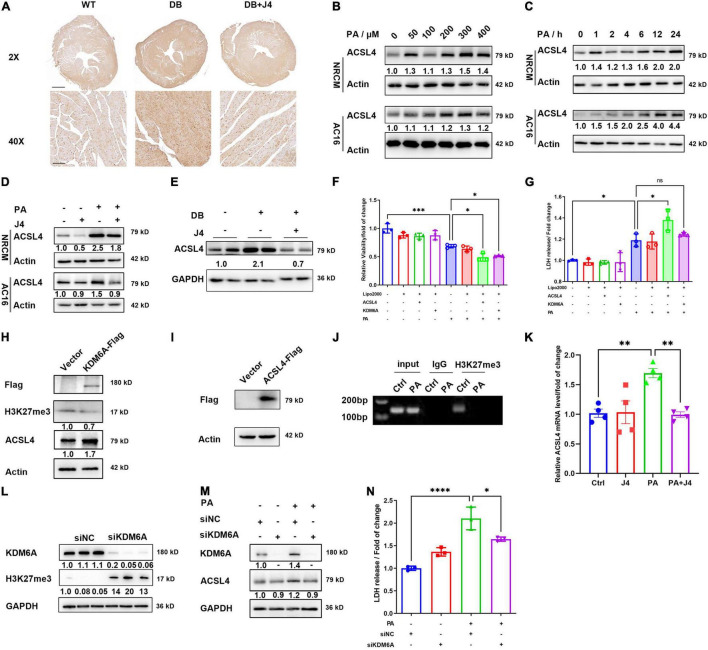
GSK-J4 reduces ACSL4 expression to ameliorate ferroptosis. **(A)** Immunohistochemistry image of 4-HNE in the cardiac section, the upper scale bar is 500 μm, while the lower scale bar is 20 μm. **(B)** Representative Western blot image of ACSL4 protein level in NRCM and AC16 cells treated with different PA concentrations. **(C)** Representative Western blot image of ACSL4 protein level in NRCM and AC16 cells treated with 400 μM PA at different time points. **(D)** Representative Western blot image shows that GSK-J4 reduces ACSL4 levels in NRCM and AC16 cells. **(E)** Representative Western blot image shows that GSK-J4 reduces ACSL4 level in an animal model. **(F,G)** Transfection of AC16 cells with KDM6A and ACSL4 exaggerates PA-induced cell viability reduction **(F)** and LDH releasement **(G)**, indicating severer cell injury. **(H)** Representative Western blot image of AC16 cells transfecting with KDM6A-flag and ACSL4 is upregulated. **(I)** Representative Western blot image of AC16 cells transfecting with ACSL4-flag. **(J)** ChIP-PCR analysis shows less H3K27me3 level at the transcription start site of ACSL4. **(K)** mRNA level of ACSL4 in AC16 cells after PA stimulation and GSK-J4 treatment. **(L)** Representative Western blot image of AC16 cells transfecting with siKDM6A or siNC. After silencing KDM6A, the level of H3K27me3 is upregulated. **(M)** Representative Western blot image of AC16 cells transfecting with siKDM6A and stimulated with 400 μM PA. After silencing KDM6A, the protein level of ACSL4 is not upregulated by PA. **(N)** Knocking down KDM6A reduces LDH releasement under PA stimulation. *P < 0.05, ^**^P < 0.01, ^***^P < 0.001, ^****^P < 0.0001, all the data significance was analyzed by ANOVA.

As shown in Figure 4, PA could trigger ferroptosis in cardiomyocytes. We also stained 4-HNE, another important biomarker of lipid peroxidation (29), in cardiac tissue by immunohistochemistry and found elevated 4-HNE in DB/DB mice. GSK-J4 reduced 4-HNE in cardiac tissue (Figure 5A). Ferroptosis is regulated by various key enzymes including ACLS4, which is pro-ferroptosis by determining the membrane lipid composition to affect the cell’s sensitivity to ferroptosis. We found that PA could dose-dependently and time-dependently upregulate ACSL4 in NRCM and AC16 cell lines (Figures 5B,C). Importantly, GSK-J4 could reserve the upregulation of ACLS4 both in the cell model (Figure 5D) and in an animal model (Figure 5E), which was consistent with its anti-ferroptosis feature.

We next asked whether GSK-J4 inhibited ACSL4 expression *via* KDM6A. We constructed KDM6A-Flag and ACSL4-Flag overexpression vector and transfected it into AC16 cell lines (Figures 5H,I), and found that after overexpressing KDM6A, the protein level of ACSL4 was also significantly upregulated (Figure 5H). Meanwhile, both overexpression of KDM6A and ACSL4 could amplify the PA-induced cardiomyocyte injury as evidenced by the LDH release assay (Figure 5F) and CCK8 cell viability assay (Figure 5G). This further support the idea that KDM6A could promote ACSL4 expression and augment lipotoxicity. We next identified that under PA stimulation, the H3K27me3 level was reduced at the transcription start site of ACSL4 by ChIP-PCR analysis (Figure 5J). The mRNA level of ACSL4 was significantly elevated by PA and reversed by GSK-J4 (Figure 5K).

One of the concerns of using small molecule antagonists is the possibility of being off-target. Hence, we applied siRNA to silence the protein expression of KDM6A and found that siKDM6A could significantly upregulate the level of H3K27me3 (Figure 5L). After silencing KDM6A, the protein level of ACSL4 did not upregulate after PA stimulation (Figure 5M), and the LDH releasement was also reversed by silencing KDM6A (Figure 5N), suggesting that reducing KDM6A indeed alleviated PA-induced cell injury.

Taken together, these data indicated that GSK-J4 could preserve H3K27me3 level and suppress ACSL4 transcription to reduce its protein level, and hence inhibit ferroptosis.

## Discussion

Epigenetic regulation is a fundamental regulatory mechanism in different diseases, which is considered as regulation of targeted genes without affecting their gene sequence. Modification of DNA or histone, as well as the participation of non-coding RNAs, are the principal paradigm of epigenetic regulation (16). Numerous reports have already reported the involvement of non-coding RNAs in the regulation of diabetes and its complications (15). However, the contribution of histone post-translational modifications and their corresponding epigenetic regulation mechanism to the development of diabetic complications is far from fully revealed. In our current work, we identified that the inhibitor of histone lysine demethylase KDM6A, GSK-J4, was capable of ameliorating cardiomyocyte injury induced by lipotoxicity under diabetes. These findings indicate that targeting epigenetic targets such as KDM6A is a promising way to attenuate diabetic complications.

GSK-J4 is a dual inhibitor for KDM6A and KDM6B, both of which are demethylases for the H3K27me3 epigenetic marker. We found that KDM6B was unaltered, while KDM6A was upregulated after PA stimulation in AC16 cells and a clear dose-dependent relationship between KDM6A and GSK-J4 in both AC16 and primary cardiomyocyte was found. Hence, KDM6A may be the major responder to GSK-J4 in cardiac tissue. Most of the research regarding GSK-J4 are focusing on its anti-cancer effects (30) by mitigating proliferation (31, 32) or attenuating metastasis (33). There are also a few reports indicating that GSK-J4 may be effective in treating diabetic nephropathy (26, 34), one of the common diabetic complications, by reducing inflammation and DNA damage or regulating E-cadherin. Hence, these evidences indicate that GSK-J4 is a potential anti-diabetic drug, which is worthy of further investigation.

H3K27me3 is considered a suppressive epigenetic marker silencing downstream genes (35, 36). As an epigenetic marker, H3K27me3 has a series of writer and eraser, including histone-lysine N-methyltransferases EZHs as writer and KDM6A/6B as an eraser, both of which are found to have abnormal expression and cause dysregulation of H3K27me3 in different diabetic complications. Liu et al. reported that H3K27me3 participated in the regulation of insulin transcription by recruiting YY1 in pancreatic beta cell (37). Thakar et al. otherwise found that more EZH2 were nuclear-translocated under high glucose stimulation and thereby inducing H3K27me3 upregulation to suppress KLF2, ultimately causing endothelial inflammation (38). These clues suggest that one epigenetic marker may exhibit totally oppose pattern and function in different tissues or disease states. It is also astonishing to find that H3K27me3 is reported to be a transcriptional-active marker for SESN2 expression in the doxorubicin-induced cardiomyopathy (39). This reveals the complexity of regulation *via* epigenetic regulations. One of the reasons for this complexity is the uncertainty of the corresponding reader protein of the exact epigenetic marker, which may be various in different organs or cells. In our current work, we found that H3K27me3 was downregulated in diabetic mice heart tissue or PA-stimulated primary cardiomyocyte or cell lines, and maintaining its level by GSK-J4 showed a significant protective effect. However, one of the major limitations of our research is that we did not get a comprehensive landscape of H3K27me3 under PA stimulation, which may help identify vital genes participating in lipotoxicity to cardiomyocytes. According to our results, we gave evidence that upregulation of ACSL4 by H3K27me3 downregulation may be involved in the PA-induced cardiomyocyte injury through initiating ferroptosis.

Ferroptosis is a newly defined cell death pathway that plays an important role in cancer therapy (40), neuron degeneration diseases (41), and chemotherapy-induced cardiomyopathy (21). Ferroptosis is characterized by lipid peroxidation and consequently membrane impairment, which ultimately leads to cell death (24, 29). Due to the significant attenuation of ERS by GSK-J4, we first considered whether the protective effect of GSK-J4 relied on the attenuation of ERS-induced cell apoptosis, as numerous reports have already revealed the role of apoptosis in the development of diabetic cardiomyopathy. However, we did not find any relief of apoptosis by GSK-J4, except for the significant remission of ROS as well as the reduction of intracellular triglyceride accumulation. Considering the core pathophysiological feature of ferroptosis is lipid peroxidation, we then analyzed whether GSK-J4 attenuated ferroptosis. Indeed, a decrease in MDA and 4-HNE levels, byproducts of lipid peroxidation, and reduction of BODIPY-C11-stained lipid peroxidation in AC16 by GSK-J4 strongly supported the idea that GSK-J4 could inhibit ferroptosis under diabetic condition. There are still no reports indicating the relationship between GSK-J4 and ferroptosis, while our data can provide a new insight into the working mechanism of GSK-J4.

ACSL4 is a kind of fatty-acid-CoA ligases and is pro-ferroptosis by shaping cellular lipid composition (42). Interfering ACSL4 protein level either by pharmacological inhibitor (43, 44) or by intrinsic active factors (45) is involved in ferroptosis in diabetic complications. Consistent with previous reports, we found significant ACSL4 elevation both *in vivo* and *in vitro*, and GSK-J4 could reverse its elevation. Overexpression of KDM6A directly led to upregulation of ACSL4, as well as exacerbation of PA-induced cardiomyocyte injury (upregulation of LDH release and downregulation of cell viability). Reduced H3K27me3 level at the transcriptional start site of ACSL4 by PA stimulation further supports the hypothesis that KDM6A could induce ACSL4 transcription by demethylating H3K27me3, and GSK-J4 could inhibit KDM6A to attenuate ACSL4 upregulation. In contrast, silencing KDM6A could alleviate PA-induced cardiomyocyte injury as evidenced by reduced LDH releasement. However, the concern may raise that silencing KDM6A also led to slightly upregulated LDH (Figure 5N). This may be explainable that KDM6A is a vital gene, which not only regulates the expression of ACLS4 but also a series of other genes. Hence, complete silencing of its expression may be somewhat detrimental to cell viability. Therefore, using GSK-J4 to suppress its activity but not directly silencing its expression may be more appropriate.

However, there are still several limitations of our work. The major limitation is that we did not get a comprehensive landscape of H3K27me3 at the whole genome under PA stimulation. Further, ChIP-seq analysis may reveal the most striking changes of H3K27me3 by PA and find the most important gene(s) involved in PA-induced lipotoxicity. Another limitation is that although GSK-J4 was screened out from the ERS-related drug library and was proven to reduce ERS levels, the role of ERS in the protective effect of GSK-J4 was not fully investigated. There are several key transcriptional factors involved in ERS, such as ATF4, ATF6, CHOP, and XBP1, while silencing ATF4 did not alter the level of KDM6A under PA stimulation (data not shown), suggesting that the upregulation of KDM6A may be related to other key transcriptional factors.

In conclusion, we identified that GSK-J4 had a cardioprotective effect under diabetes conditions both *in vivo* and *in vitro* by ameliorating ferroptosis and reducing cardiomyocyte injury. GSK-J4 could preserve the H3K27me3 level and hence suppressing ACSL4 upregulation to attenuate ferroptosis. Our work suggests the participation of epigenetic regulation in the development of metabolism disorders and calls for further studies to reveal the changes of different epigenetic modification markers under diabetes.

## Materials and Methods

### Cell Culture and Treatment

AC16 cell lines were purchased from Shanghai Zhong Qiao Xin Zhou Biotechnology Company (Shanghai) with an STR identification report. AC16 cell lines were cultured with DMEM/F12 medium with 10% fetal bovine serum (CellMax, Lanzhou, China, SA311.02) and 1% penicillin/streptomycin solution (Gibco, New York, United States, 151540-122). GSK-J4 was dissolved in DMSO as a 10 mM stock solution and was administrated to cell 2 h before PA stimulation. PA was dissolved in NaOH and conjugated with 20% BSA. An equivalent amount of DMSO or BSA was given as blank control. Primary neonatal rat cardiomyocyte was separated from D1 to D7 neonatal rat. In brief, the ventricular of a neonatal rat was harvested and digested with enzyme digestion solution (2% Collagenase, Worthington, California, United States, LS004176 + 6% trypsin, BioFroxx, Einhausen, Germany, 1004GR025 + 0.1% Benzonase, Yeasan, Shanghai, China, 20125ES25, dissolved in HBSS). The ventricle was minced into small pieces and digested at 37°C for 30 min with gentle shaking. Digestion was stopped by adding 10 ml FBS into the solution and filtered with a 70 μm cell filter (BIOFIL, Guangzhou, China, CSS013070). The separated cells were collected by centrifugation at 4°C with 400 g for 10 min, and the cell pellet was washed with 1X red blood cell lysis (Servicebio, Wuhan, China, G2015) and PBS twice. The cardiomyocyte and fibroblast were separated by different attaching rates. After seeding in the culture plate for 1 h, the supernatant was collected and centrifugated to collect cardiomyocytes. The cardiomyocyte was cultured in DMEM/F12 (Servicebio, Wuhan, China, G4610) supplemented with 10% fetal bovine serum (CellMax, Lanzhou, China, SA311.02) and 1% penicillin/streptomycin solution (Gibco, New York, United States, 151540-122). The cell culture plate was pre-coated with 1% gelatin (Sigma, Darmstadt, Germany, G9382).

### Cell Viability Assay and Drug Screen

AC16 cell was cultured in a 96-well plate at 1 × 10^4^ cells per well. GSK-J4 or PA was administrated at the intended concentration shown in each experiment. DMSO or BSA was used as the control. After stimulating cells for an appropriate time, the medium was discharged and 100 μl of freshly prepared 10% CCK8 working solution (Meilunbio, Dalian, China, MA0218) was added to each well. After 30 min of incubation at 37°C, the optical density was analyzed at 450 nm. For drug screen assay, ERS Library (MCE, New Jersey, United States, HY-L054, HYCPK8142) was diluted to the intended concentration with a cell culture medium. Cells were pretreated with corresponding drugs for 2 h and 400 μM PA was administrated for 24 h. Cell viability was measured by a CCK-8 assay. Each drug was tested with five replications. The relative cell viability was calculated as follows:


$$r\,e\,l\,a\,t\,i\,v\,e\,c\,e\,l\,l\,v\,i\,a\,b\,i\,l\,i\,t\,y=\frac{\begin{array}{c} \left(O\,D\,450\,o\,f\,d\,r\,u\,g-O\,D\,450\,o\,f\,P\,A\right)\\ -O\,D\,450\,o\,f\,B\,l\,a\,n\,k \end{array}}{\begin{array}{c} \left(O\,D\,450\,o\,f\,B\,S\,A-O\,D\,450\,o\,f\,P\,A\right)\\ -O\,D\,450\,o\,f\,B\,l\,a\,n\,k \end{array}}$$

### Animal Model and GSK-J4 Administration

The 8-week-old DB/DB mice and their littermate wild-type control DB/BKS mice were purchased from Beijing Vital River Laboratory Animal Technology Company and were housed at the SPF environment at the ServiceBio biotechnology company at Wuhan. The animal experiment was carried out with the approval of the Institutional Animal Care and Use Committee of Huazhong University of Science and Technology. GSK-J4 (MCE, New Jersey, United States, HY-15648B) was dissolved in 10% DMSO (MCE, New Jersey, United States, HY-Y0320) followed by 40% PEG300 (MCE, New Jersey, United States, HY-Y0873), 5% Tween 80 (MCE, New Jersey, United States, HY-Y1891), and 45% PBS (Servicebio, Wuhan, China, G4200) to form a clear yellow solution. GSK-J4 was injected intraperitoneally at the dosage of 10 mg/kg every 2 days. An equivalent volume of vector solution was injected into DB/DB and DB/BKS mice as control.

### Protein Extraction and Western Blot

Protein of cell or cardiac tissue was extracted as mentioned before. In brief, after collecting cell pellet or weighting cardiac tissue, they were lysed with RIPA lysis buffer (Servicebio, Wuhan, China, G2002) supplemented by protease and phosphatase inhibitor cocktail (APEXBio, Houston, United States, K1007, K1015). After mechanical disruption, the homogenate was incubated on ice for 15 min and thoroughly vortexed every 5 min. Then, the lysis was centrifugated at 16,000 g for 15 min to remove insoluble debris. The protein concentration was measured with the BCA kit (Beyotime, Shanghai, China, P0009) and adjusted to the same. After being boiled with 5X protein loading buffer (Beyotime, Shanghai, China, P0015L) at 100°C for 10 min, 20–40 μg protein were loaded into SDS-PAGE gel and electrophoresis with Tris-Glycine buffer is done. Then, the protein was transferred to a 0.45 μM PVDF membrane with 20% methanol Tris-Glycine buffer. The membrane was blocked with 5% BSA in TBST, and the corresponding primary antibody was incubated at 4°C overnight. HRP-conjugated secondary antibody was used to detect binding primary antibody with ECL kit (Abclonal, Wuhan, China, RM00021). All the Western blots were conducted at least three times to confirm their reproducibility. Following antibodies were used at the intended dilution: H3K27me3 (Abclonal, Wuhan, China, A2363, 1:1,000), KDM6A (CST, Massachusetts, United States, 33510S, 1:1,000), KDM6B (Abclonal, Wuhan, China, A17382, 1:1,000), Flag (Abclonal, Wuhan, China, AE005, 1:1,000), GAPDH (Abclonal, Wuhan, China, AC033, 1:10,000), BIP (Abclonal, Wuhan, China, A0241, 1:1,000), ATF4 (Abclonal, Wuhan, China, A0201, 1:1,000), ATF6 (Abcam, Cambridge, United States, Ab203119, 1:1,000), Caspase-3 (CST, Massachusetts, United States, 9662S, 1:1,000), Caspase-9 (CST, Massachusetts, United States, 9508S, 1:1,000), Caspase-12 (CST, Massachusetts, United States, 2202S, 1:1,000), ACSL4 (Abclonal, Wuhan, China, A6826, 1:1,000), and β-actin (Abclonal, Wuhan, China, AC026, 1:10,000).

### Lactate Dehydratase Assay

After proper treatments, the culture medium of each well was collected and centrifugated at 500 g for 5 min to remove any cell debris. The supernatant was then transferred to a new Ep tube and measured the LDH level according to the manufacturer’s protocol (Beyotime, Shanghai, China, C0016). The optical density was normalized according to the control group.

### Annexin V/PI Cell Apoptosis Analysis

Cell was collected by trypsin digestion and washed with PBS twice. The cell was stained according to the manufacturer’s protocol (Vazyme, Nanjing, China, A211-01) and analyzed by flow cytometry (Accuri, MI, United States).

### Immunofluorescence and Immunohistochemistry

Cell or paraffin embedding section immunofluorescence was carried out as previously described. After fixation, permeabilization, antigen retrieval and blocking, and cell or section were incubated with corresponding primary antibodies at 4°C overnight. On the second day, the cell or section was washed with PBS three times, and Cy3- and Alexa Fluor 488-conjugated secondary antibodies were used for immunofluorescence or HRP-conjugated secondary antibody was used for immunohistochemistry. DAB was used to detect the primary antibody. The nuclear was stained with DAPI for immunofluorescence or hematoxylin for immunohistochemistry. The fluorescence images were taken with confocal microscopy (Nikon, Tokyo, Japan). The DAB-stained cardiac section was scanned by Pannoramic MIDI (3DHISTECH, Hungary). Fluorescence intensity was analyzed by ImageJ and normalized to control. Following antibodies were used at the intended dilution: H3K27me3 (Abclonal, Wuhan, China, A2363, 1:200), cTnI (ProteinTech, Wuhan, China, 66376-1-Ig, 1:200), 4-HNE (Bioss, Beijing, China, bs-6313R, 1:200), Cy3-conjugated goat-anti-rabbit secondary antibody (ServiceBio, Wuhan, China, GB21303, 1:200), and Alexa Fluro 488-conjugated goat-anti-mouse secondary antibody (ServiceBio, Wuhan, China, GB25301, 1:200).

### mRNA Extraction, Reverse Transcription PCR, and Real-Time PCR

Cell mRNA was extracted by Trizol (Vazyme, Nanjing, China, R401-01) and reverse transcripted according to the manufacturer’s protocol (Yeasan, Shanghai, China, 11141ES60). The mRNA levels of KDM6A and KDM6B were quantified by SYBR-green real-time PCR (Yeasan, Shanghai, China, 11201ES03) on CFX96 (Bio-Rad, California, United States) with the following primers:

KDM6A-F/R: TTCCTCGGAAGGTGCTATTCA/GAGGCTG GTTGCAGGATTCA;

KDM6B-F: CACCCCAGCAAACCATATTATGC/CACACAG CCATGCAGGGATT.

The relative mRNA level was calculated by the 2^–ΔΔ*Ct*^ method.

### Triglyceride, Cholesterol, and Blood Glucose Measurement

Serum was collected by centrifugating coagulant blood at 3,500 rpm for 15 min. Serum triglyceride, cholesterol, and blood glucose were measured according to the manufacturer’s protocol (Nanjing Jiancheng Bioengineering Institute, Nanjing, China, A110 for triglyceride, A111 for cholesterol, F006 for blood glucose). Triglyceride and cholesterol measurement of tissue homogenate or cell pellet were also conducted with a few adaptations. Cardiac tissue or cell pellet was milled by PBS supplemented with protease inhibitor, and the homogenate was measured by the kit. The triglyceride and cholesterol levels were adjusted to the protein concentration of the homogenate.

### Malondialdehyde Measurement

Serum MDA level or AC16 cell pellet MDA level was measured by MDA assay kit according to the manufacturer’s instruction (Nanjing Jiancheng Bioengineering Institute, Nanjing, China, A003). The cell MDA measurement was adjusted to protein concentration.

### Reactive Oxygen Species Measurement

Intracellular ROS level was measured by two different ROS probes, DHE (Beyotime, Shanghai, China, S0063) and DCFH (Beyotime, Shanghai, China, S0033S). DHE was analyzed by flow cytometric with 1 × 10^4^ events in each group. DCFH was analyzed by fluorescence microscopy. The fluorescence intensity of DCFH was analyzed by ImageJ and normalized to the control group. About 200 cells were analyzed in each group.

### Transmission Electrical Microscopy

After proper treatments, the medium was discharged and AC16 cells were immediately fixed with pre-cooled 2.5% glutaraldehyde at 4°C for 30 min. Then, cells were collected with a soft scraper and centrifugated to collect the cell pellet. The 2.5% glutaraldehyde was refreshed and the cell was further fixed for 24 h at 4°C. The following protocol of transmission electrical microscopy was conducted by ServiceBio (Wuhan), and the images were taken by HITACHI, Tokyo, Japan HT7700 120 kv.

### C11-BODIPY Staining and Fluorescence Microscopy

C11-BODIPY (Abclonal, Wuhan, China, RM02821) was dissolved by DMSO as 10 mM stock (1000X). The working concentration of C11-BODIPY was 10 μM, diluted by a cell culture medium. Cells were washed with pre-warmed PBS twice and stained with a C11-BODIPY working solution at 37°C for 30 min. Then, the cells were observed and taken photos by Nikon confocal microscopy at 488 nm (for oxidated lipid) and 561 nm (for reduced lipid) excitation length. The fluorescence intensity of each cell was measured by ImageJ to calculate the ratio of red to green signal. About 50 cells in each group were analyzed.

### Plasmid Construction and Transfection of Plasmid or siRNA

The total RNA of the AC16 cell was extracted as mentioned earlier and was reverse-transcripted by the oligo-dT method (Vazyme, Nanjing, China, R211-01). The CDS of *homo sapiens* ACSL4 and KDM6A were amplified by the following primers:

KDM6A-F: tccagtgtggtggaattcATGAAATCCTGCGGAGTGT CG,

KDM6A-R: atcgtcatccttgtagtcAGATGAGGCGGATGGTAAT GG;

ACSL4-F: agtgtggtggaattcATGAAACTTAAGCTAAATGTGC TCACCA;

ACSL4-R: gtcatccttgtagtcTTTGCCCCCATACATTCGTTCA ATGT.

The pcDNA3.1-flag vector was linearized by the following primers:

Flag-F: gactacaaggatgacgatGACAAGG; Flag-R: gaattccaccaca ctggaCTAGTGG.

The lowercase represents the homogenous ligation sequence. DNA fragments were recycled from agarose gel according to the manufacturer’s instructions (Yeasan, Shanghai, China, 19101ES50). The insertion fragment and linearized vector were ligated by homologous recombinase according to the manufacturer’s protocol (Vazyme, Nanjing, China, C112-01) and transformed into DH5α. The amplified plasmid was extracted (Vazyme, Nanjing, China, DC201-01) and 2 ug plasmid was transfected into AC16 cell by HighGene Transfection reagent (Abclonal, Wuhan, China, RM09014). After transfection for 24 h, the cells were harvested or used for downstream examination.

To silence the expression of KDM6A, we designed a pair of siRNA sequences targeting *Homo sapiens* KDM6A (GeneID: 7403) with two bases of thymidine overhang at 3′ end:

GCAGAGGAGCCGUGGAAAAtt, UUUUCCACGGCUCCU CUGCtt.

And the sequence of negative control of siRNA was:

UUCUCCGAACGUGUCACGUtt, ACGUGACACGUUCGG AGAAtt.

After 5*10^5^ AC16 attaching to the 6-well plate, 0.1 nmol of siRNA together with 5 μl transfection reagent (Yeasan, Shanghai, China, 40806ES01) were used according to the manufacturer’s protocol. After 24 h of transfection, PA was added into the medium for another 24 h.

### ChIP

ChIP was conducted according to the manufacturer’s protocol (Beyotime, Shanghai, China, P2078) with some adaptation. In brief, 5 × 10^6^ cells were fixed with 3% formaldehyde, and the nucleosome was digested by micrococcal nuclease (Thermo, Massachusetts, United States, EN0181). A 2.5 ug H3K27me3 primary antibody (Abclonal, Wuhan, China, A2363) and isotype control IgG (Abclonal, Wuhan, China, AC005) were used to precipitate. After eluting DNA by DNA recycle kit (Beyotime, Shanghai, China, D0033), the following primers were used to detect the precipitated DNA: forward: CGTCAGTCGAAAAGGCGGAG; reverse: CGAGACTGACAGAAGCGGAT. The product length was 128 bp, located at chrX:109733444–109733571, where the 3′ flank of ACSL4 is present.

## Data Availability Statement

The original contributions presented in the study are included in the article/supplementary material, further inquiries can be directed to the corresponding author/s.

## Ethics Statement

The animal study was reviewed and approved by the Institutional of Animal Care and Use Committee of Huazhong University of Science and Technology.

## Author Contributions

KX and XL carried out the mice experiment under animal experimentation agreement number 2019005. KX, BW, and XH performed cell culture and biochemistry assay. WH, YL, and WZ carried out the histology assay and immunofluorescence assay. WH drafted the manuscript. LC, WH, and JC participated in the design and preparation of the manuscript, as well as conceived the study and participated in the coordination and preparation of the manuscript. All authors read and approved the final draft.

## Conflict of Interest

The authors declare that the research was conducted in the absence of any commercial or financial relationships that could be construed as a potential conflict of interest.

## Publisher’s Note

All claims expressed in this article are solely those of the authors and do not necessarily represent those of their affiliated organizations, or those of the publisher, the editors and the reviewers. Any product that may be evaluated in this article, or claim that may be made by its manufacturer, is not guaranteed or endorsed by the publisher.
